# UHPLC-QTOF-MS Profiling of Chemical Constituents in POW9^TM^ Cocktail with Antioxidant and Anti-Proliferative Potentials Against Vero, MCF-7 and MDA-MB-231 Cells

**DOI:** 10.3390/ijms27031246

**Published:** 2026-01-27

**Authors:** Chirra Taworntawat, Pisit Tonkittirattanakul, Pongpisid Koonyosying, David D. Perrodin, Somdet Srichairatanakool, Wachiraporn Tipsuwan

**Affiliations:** 1 G&B Enzymes Company Limited, Bang Bon Subdistrict, Bang Bon District, Bangkok 10150, Thailand; suarkom@gmail.com (C.T.); iam-gig@hotmail.com (P.T.); 2 Faculty of Veterinary Medicine, Chiang Mai University, Chiang Mai 50100, Thailand; pongpisid.koo@cmu.ac.th; 3 Institute for Population and Social Research, Mahidol University, Nakhon Pathom 73170, Thailand; davidd.per@mahidol.ac.th; 4 Department of Biochemistry, Faculty of Medicine, Chiang Mai University, Chiang Mai 50200, Thailand; 5 Division of Biochemistry, School of Medical Sciences, University of Phayao, Phayao 56000, Thailand

**Keywords:** antioxidant, breast cancer, UHPLC-ESI-QTOF-MS, MCF-7, MDA-MB-231, phenolics, cytotoxicity

## Abstract

Breast cancer remains one of the most prevalent and lethal malignancies affecting women worldwide, underscoring the need for safer and more effective therapeutic strategies. This study investigated the phytochemical composition, antioxidant activity, and antiproliferative potential of POW9™, a proprietary botanical blend formulated from nine medicinal plant extracts. Comprehensive phytochemical profiling was performed using ultrahigh-performance liquid chromatography coupled with electrospray ionization quadrupole time-of-flight mass spectrometry (UHPLC-ESI-QTOF-MS) in both positive and negative ionization modes. A total of 34 compounds were identified in negative mode and 27 compounds in positive mode, comprising flavonoids, terpenoids, steroids, organic acids, peptides, glycosides, and lipids. POW9™ exhibited high total phenolic content (190.3 ± 3.5 mg gallic acid equivalents/g) and total flavonoid content (115.2 ± 1.5 mg quercetin equivalents/g), along with strong antioxidant activity, demonstrated by a 2,2-diphenyl-1-picrylhydrazyl (DPPH) radical scavenging with a half-maximal inhibitory concentration (IC_50_) of 1.66 mg/mL (33.73 mg Trolox equivalents/g). Cytotoxicity assessment revealed minimal toxicity toward normal Vero cells. In contrast, POW9™ significantly inhibited the proliferation of human breast cancer cell lines in a concentration- and time-dependent manner. The IC_50_ values were 6.75 mg/mL for MCF-7 cells and 18.08 mg/mL for MDA-MB-231 cells after 72 h of treatment, while prolonged exposure (96 h) further enhanced antiproliferative efficacy, reducing the IC_50_ to 2.34 mg/mL. These findings demonstrate that POW9™ is a chemically diverse herbal formulation with potent antioxidant and selective anti-breast cancer activities, supporting its potential development as a complementary therapeutic or nutraceutical agent for breast cancer management.

## 1. Introduction

Breast cancer is one of the most prevalent malignancies and a leading cause of cancer-related mortality among women worldwide, representing a major global health burden [1,2,3,4]. However, advances in early detection and treatment have improved survival rates, significant clinical challenges remain, including disease recurrence, therapeutic resistance, systemic toxicity, and limited treatment options for aggressive subtypes such as triple-negative breast cancer (TNBC) [5,6,7,8,9]. Current treatment strategies include surgery, chemotherapy, hormone therapy, and targeted agents. For example, endocrine therapies for estrogen receptor (ER)-positive tumors, human epidermal growth factor receptor 2 (HER2)-directed agents (such as trastuzumab and related monoclonal antibodies) for HER2-overexpressing breast cancers, and other molecularly targeted approaches aim at specific signaling pathways involved in tumor growth and survival. While ER-positive and HER2-positive breast cancers benefit from targeted therapies, TNBC lacks specific molecular targets and is associated with poor prognosis and limited therapeutic options [7,10]. These limitations underscore the need for safer, more effective, and complementary therapeutic strategies with improved clinical relevance. Medicinal plants and natural products have long been recognized as rich sources of bioactive compounds, including phenolics, flavonoids, terpenoids, and fatty acids, many of which exhibit antioxidant, anti-inflammatory, and anticancer activities [10,11,12,13,14,15,16,17,18]. Increasing attention has been toward multi-component botanical formulations, which may offer enhanced therapeutic efficacy through multi-targets and potentially synergistic mechanisms while minimizing toxicity toward normal cells. Such formulations are particularly attractive as adjunct or nutraceutical approaches, supporting long-term disease management and improving patient quality of life. From a sustainability perspective, plant-based products represent renewable and accessible resources that align with current trends in integrative, preventive, and personalized medicine.

Despite growing interest in botanical formulations, many products remain insufficiently characterized with respect to their chemical composition and biological activity, limiting their translational value and clinical applicability. Advanced analytical platforms, particularly ultrahigh-performance liquid chromatography coupled with UHPLC-ESI-QTOF-MS enable comprehensive, untargeted profiling of complex botanical products, facilitating the identification of diverse phytochemical constituents [19,20]. Such analytical strategies are essential for ensuring quality control, reproducibility, and mechanistic insight—key requirements for translational research and future clinical development of botanical-based interventions.

POW9™ is a proprietary botanical blend formulation developed by G&B Enzymes Company Limited (Bangkok, Thailand), composed of extracts derived from nine medicinal plants traditionally associated with antioxidant and health-promoting properties. The formulation was designed as an antioxidant-rich nutraceutical intended to support general health and wellness, with potential complementary relevance to oxidative stress–related conditions, including cancer. The biological rationale for POW9™ is based on the complementary and potentially synergistic actions of its constituent plant extracts, which are rich in phenolics, flavonoids, terpenoids, fatty acids, and other bioactive metabolites known to modulate redox balance, inflammatory processes, and cancer-related cellular pathways. Despite its commercial availability, the detailed phytochemical composition and anticancer-related biological activities of POW9™ have not been systematically characterized, underscoring the need for comprehensive chemical profiling and biological evaluation. This study integrates untargeted UHPLC-ESI-QTOF-MS profiling with antioxidant evaluation and comparative antiproliferative assessment of POW9™ in hormone-dependent (MCF-7) and hormone-independent (MDA-MB-231) breast cancer cell lines, along with selectivity toward normal Vero cells. This combined approach provides clinically relevant insight and highlights the translational value of POW9™ as a complementary or nutraceutical candidate for breast cancer management.

## 2. Results

### 2.1. UHPLC-ESI-QTOF-MS Based Phytochemical Profiling

**Figure 1:**
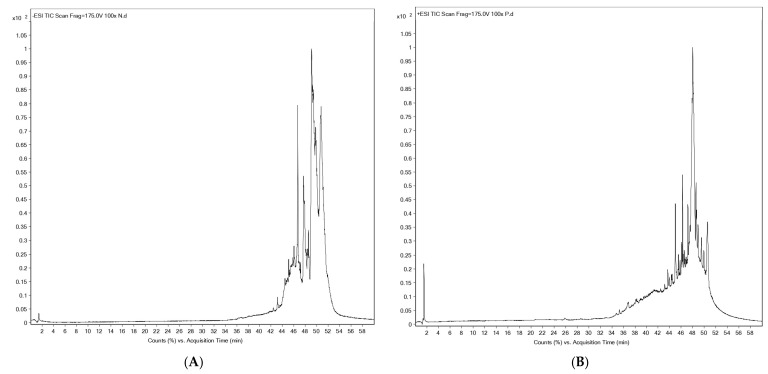
Profiles of phenolic compounds from POW9^TM^ analyzed with UHPLC-ESI-QTOF-MS using negative mode (**A**) and positive mode (**B**).

The phytochemical composition of POW9™ was analyzed using UHPLC-ESI-QTOF-MS in both negative and positive ionization modes. The base peak chromatograms demonstrated well-resolved peaks across a 60-minute elution period. In the negative ion mode (Figure 1A and Table 1A), a total of 34 compounds were identified, predominantly consisting of esters and carboxylic acids. Notable compounds included butyl butyryl lactate, ricinoleic acid, glyceryl monoleate, octadecadienoic acid, and eicosanedioic acid.

Positive ion mode analysis (Figure 1B and Table 1B) resulted in the identification of 27 compounds, mainly phospholipids, peptides, glycosides, and bioactive secondary metabolites. Key compounds included phosphatidic acid, phosphoethanolamine derivatives, Trp-His-Val, Thr-Thr, ceramide glycosides, and tafluprost. The combined ionization approaches provided a comprehensive phytochemical profile of POW9™, highlighting its chemical diversity and complexity.

### 2.2. Total Phenolic and Flavonoid Content, and Antioxidant Activity

**Figure 2:**
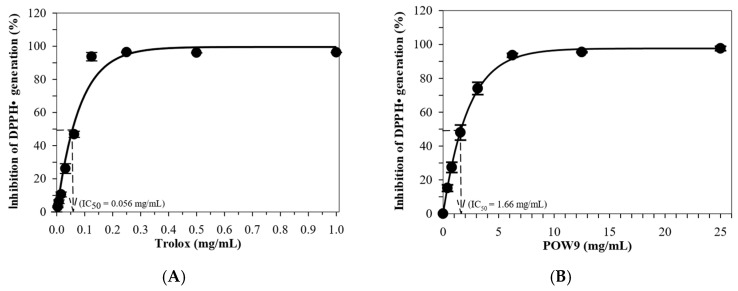
Inhibition of DPPH radical (DPPH•) generation by Trolox (**A**) and POW9^TM^ (**B**). Data are expressed in mean ± standard error of the mean (SEM) of three independent experiments.

The antioxidant properties of POW9™ were assessed through the determination of total phenolic content (TPC), total flavonoid content (TFC), and DPPH radical scavenging activity. POW9™ exhibited a TPC of 190.3 ± 3.5 mg GAE/g and a TFC of 115.2 ± 1.5 mg quercetin equivalent (QE)/g (Table 2). The DPPH assay demonstrated that both POW9™ and Trolox inhibited free radical generation in a concentration-dependent manner (Figure 2). The IC_50_ values were calculated by nonlinear regression analysis: 1.66 mg/mL for POW9™ and 0.056 mg/mL for Trolox. The antioxidant capacity of POW9™ was equivalent to 33.73 mg Trolox per gram, corresponding to 20.24 mg Trolox-equivalent antioxidant activity (TEAC) per 600 mg capsule, indicating its substantial antioxidant activity under the tested conditions.

### 2.3. Effect of POW9^TM^ on Vero Cell Viability

**Figure 3:**
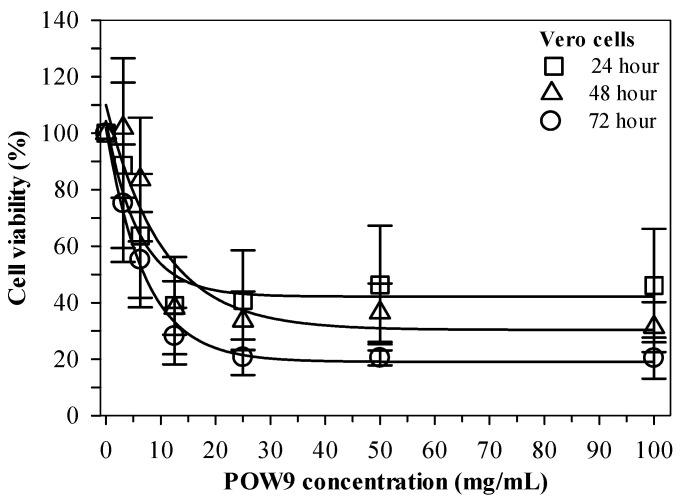
Viability of Vera cells treated with POW9^TM^ (0–100 mg/mL) for 24, 48 and 72 h. Data obtained from three independent experiments are expressed as mean ± SD.

The cytotoxic potential of POW9™ was assessed using a cell viability assay in Vero cells treated with concentrations ranging from 0 to 100 mg/mL for 24, 48, and 72 h. Microscopic examination did not reveal overt morphological disruption of Vero cells at low to moderate concentrations of POW9™. At higher concentrations, although changes in culture medium appearance were observed, quantitative 3-(4,5-dimethylthiazol-2-yl)-2,5-diphenyltetrazolium bromide (MTT) analysis indicated that overall cell viability remained above 50% under the experimental conditions (Figure S1). Quantitative analysis demonstrated that POW9™ maintained high cell viability across all tested time points (Figure 3). These results indicate that POW9™ exhibits relatively low cytotoxicity toward Vero cells compared with its effect on breast cancer cell lines under the experimental conditions.

### 2.4. Antiproliferative Effect of POW9™ on Breast Cancer Cells

**Figure 4:**
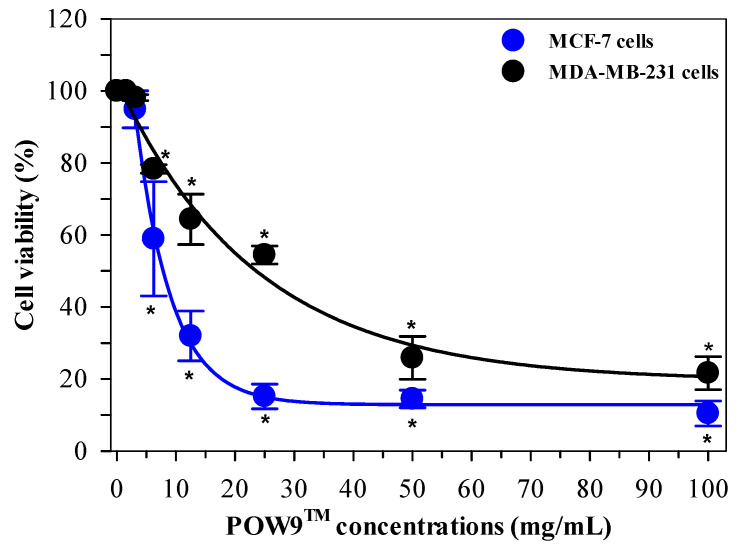
Dose-dependent antiproliferative effects of POW9^TM^ on breast cancer cell lines. MCF-7 and MDA-MB-231 cells were treated with POW9^TM^ (0–100 mg/mL) for 72 h. Cell viability was assessed using the MTT assay and expressed as mean ± SD of three independent experiments. Statistical analysis was performed using two-way ANOVA followed by Tukey’s post hoc test. * *p* < 0.05 versus untreated control.

The antiproliferative activity of POW9™ was evaluated using MCF-7 and MDA-MB-231 breast cancer cell lines treated with concentrations ranging from 0 to 100 mg/mL for 72 h. Microscopic examination revealed a marked reduction in cell density at higher concentrations, accompanied by visible morphological alterations (Figure S2). MTT assay results demonstrated that POW9™ significantly reduced cell viability in a concentration-dependent manner in both cell lines. In this study, the IC_50_ value represents the primary measure of antiproliferative potency. POW9™ was more effective against MCF-7 cells, with an IC_50_ value of 7.81 mg/mL, compared to 23.6 mg/mL for MDA-MB-231 cells. At 100 mg/mL, POW9™ inhibited approximately 90% of MCF-7 cell growth and 70% of MDA-MB-231 cell growth, indicating pronounced antiproliferative effects in vitro (Figure 4).

**Figure 5:**
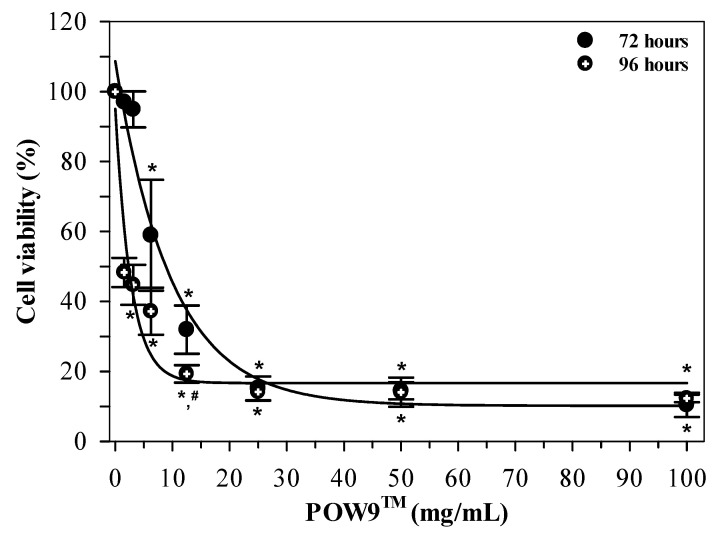
Time-dependent effects of POW9^TM^ on MDA-MB-231 cell viability. Cells were treated with POW9^TM^ (0–100 mg/mL) for 72 and 96 h. Cell viability was determined using the MTT assay and expressed as mean ± SD of three independent experiments. Statistical significance between durations at the same concentration was assessed using two-way ANOVA followed by Tukey’s post hoc test. * *p* < 0.05 versus untreated control (same time), ^#^ *p* < 0.05 versus 72 h at same concentration.

To assess the effect of prolonged exposure, MDA-MB-231 cells were treated with POW9™ (0–100 mg/mL) for 72 and 96 h. Microscopic analysis revealed a progressive reduction in cell density with increasing concentrations (Figure S3). As shown in Figure 5, POW9™ treatment resulted in a concentration-dependent reduction in cell viability at both time points. Notably, extended exposure for 96 h led to a more pronounced decrease in cell viability compared to 72 h at corresponding concentrations. A significant decrease in cell viability was found at low concentrations (1.56–3.12 mg/mL), yielding IC_50_ values of 2.34 mg/mL for 96 h treatment, whereas no substantial additional reduction in viability was observed at higher concentrations, suggesting no further reduction in viability at higher concentrations under the tested conditions. Thus, extended treatment duration was associated with enhanced antiproliferative activity of POW9™, as reflected by reduced IC_50_ values at 96 h compared with 72 h.

Taken together, these findings demonstrate that the anticancer efficacy of POW9™ is both dose- and time-dependent.

## 3. Discussion

The present study provides a comprehensive evaluation of the phytochemical composition, antioxidant activity, and antiproliferative potential of POW9™, a multi-component botanical formulation. Using UHPLC-ESI-QTOF-MS operated in both negative and positive ionization modes, POW9™ was shown to possess a chemically diverse profile comprising fatty acids, organic acids, terpenoids, glycosides, peptides, phospholipids, and other secondary metabolites [19,20,21,22,23]. The higher number of compounds detected in negative ionization mode is consistent with the prevalence of acidic, phenolic, and lipid-associated constituents commonly found in plant-derived extracts and complex botanical matrices [19,21]. Several identified compounds associated with antioxidant, anti-inflammatory and anticancer activities, including hydroxy- and dihydroxy-octadecenoic acids, ricinoleic acid, and octadecadienoic acid have been reported to modulate oxidative stress and inhibit cancer cell proliferation [24,25,26,27]. Terpenoid- and phenolic-related constituents, such as blumenol derivatives and citronellol acetate, are also known for their radical scavenging and anti-inflammatory properties [28,29]. Although the contribution of individual compounds cannot be conclusively delineated due to the complexity of the formulation, the observed biological effects are likely mediated through additive or synergistic interactions among these constituents.

The antioxidant assays performed in this study (TPC, TFC, and TEAC) were conducted on the crude POW9™ formulation and were intended solely to provide general compositional and chemical characterization. These assays were not designed to assess intracellular antioxidant effects or to establish a mechanistic link between redox modulation and antiproliferative activity. As such, the antioxidant measurements should not be interpreted as evidence of anticancer mechanisms, nor should they be directly comparable to reference compounds with established anticancer efficacy. Consistent with its phytochemical richness, POW9™ exhibited strong antioxidant capacity, as evidenced by high total phenolic and flavonoid contents and potent DPPH radical scavenging activity. The relationship between phenolic and flavonoid abundance and antioxidant efficacy has been widely documented for botanical extracts [29,30,31,32]. Importantly, the antioxidant-effective concentration was substantially lower than the concentration required to inhibit cancer cell proliferation, suggesting that POW9™ may exert protective antioxidant effects without impairing normal cellular viability.

Evaluation of cytotoxicity demonstrated that POW9™ exhibited minimal toxicity toward normal Vero cells across all tested concentrations and time points, supporting a favorable in vitro selectivity profile. In contrast, POW9™ significantly inhibited the proliferation of both MCF-7 and MDA-MB-231 breast cancer cells in a concentration- and time-dependent manner. The formulation was more potent against estrogen receptor-positive MCF-7 cells than against the more aggressive, hormone-independent MDA-MB-231 cells, a pattern that has been commonly reported and may reflect differences in hormone signaling, redox regulation, and metabolic sensitivity between these cells [9,33].

Notably, prolonged exposure markedly enhanced the antiproliferative efficacy of POW9™, particularly in MDA-MB-231 cells [9,34]. As demonstrated by comparative viability analysis, treatment for 96 h resulted in significantly greater growth inhibition than 72 h at equivalent concentrations, corresponding to a reduction in the IC_50_ value from 18.08 mg/mL to 2.34 mg/mL. Time-dependent responses of this nature are frequently observed for phytochemical-rich formulations and are often attributed to cumulative oxidative stress, delayed activation of apoptotic signaling pathways, and progressive disruption of cellular metabolic homeostasis [16,35]. The pronounced enhancement of activity in the estrogen-independent MDA-MB-231 model is particularly noteworthy given the limited therapeutic options available for triple-negative breast cancer.

The present study did not investigate molecular mechanisms of action, and no direct comparisons were made with isolated plant extracts or reference bioactive compounds. Hence, the observed antiproliferative effects should be interpreted strictly as phenomenological outcomes of exposure to a complex botanical formulation rather than as evidence of specific biochemical or signaling pathways [36]. This study combines untargeted high-resolution mass spectrometric profiling with in vitro cell viability assays to provide a descriptive characterization of a multi-component botanical formulation and its comparative antiproliferative effects in breast cancer cell models. The use of both hormone-dependent and hormone-independent breast cancer models provides broader relevance to heterogeneous breast cancer subtypes. These findings support further investigation of POW9 as a phytochemical-rich formulation with selective antiproliferative activity in vitro, while emphasizing the need for mechanistic, in vivo, and safety studies to establish translational relevance.

Despite these strengths, several limitations should be acknowledged. First, the study was conducted exclusively in vitro, and therefore, the observed biological effects may not fully reflect in vivo pharmacokinetics, bioavailability, or metabolic interactions. Second, antioxidant activity was assessed only at the formulation level and outside a cellular context, limiting its biological interpretability. Third, the antiproliferative effects were assessed using a single viability assay, and specific molecular mechanisms such as apoptosis induction, cell cycle arrest, or oxidative stress modulation were not directly investigated. Fourth, no benchmarking against isolated compounds or clinically relevant anticancer agents was performed. Consequently, the findings should be viewed as preliminary and descriptive, providing a basis for future mechanistic and comparative investigations rather than definitive evidence of anticancer mechanisms. Future studies should include formal physicochemical stability assessments to further characterize POW9™ under extended incubation conditions.

Finally, as POW9™ is a complex botanical blend, the individual contribution of specific compounds or potential synergistic interactions could not be definitively delineated. Future studies should focus on elucidating the molecular mechanisms underlying the anticancer effects of POW9™, including the involvement of apoptotic pathways, oxidative stress regulation, and hormone-related signaling. In vivo studies and pharmacokinetic evaluations are warranted to confirm efficacy, safety, and bioavailability. Additionally, fractionation studies or combination analyses may help identify key bioactive constituents and synergistic interactions, thereby facilitating optimization of the formulation for therapeutic or nutraceutical applications.

## 4. Materials and Methods

### 4.1. Chemicals, Reagents and Labware

2,2′-Azino-bis(3-ethylbenzothiazoline-6-sulfonic acid) (ABTS), dimethyl sulfoxide (DMSO), DPPH, MTT, Folin–Ciocalteu reagent, aluminum chloride, potassium acetate, sodium carbonate, Trolox (6-hydroxy-2,5,7,8-tetramethylchroman-2-carboxylic acid), Tween 20, quercetin (Q) and gallic acid (GA) standard were purchased from Sigma-Aldrich Chemicals Company Limited, Saint Louis, MO, USA. Phosphate-buffered saline pH 7.4 (PBS) solution, trypsin-ethylene diaminetetraacetic acid (T-EDTA), RPMI 1640 medium, Dulbecco’s modified Eagle medium (DMEM), fetal bovine serum (FBS), L-glutamine, penicillin and streptomycin were purchased from Hyclone^TM^ (Global Life Sciences Solutions USA LLC, Wilmington, DE, USA). Organic solvents including acetonitrile and formic acid (HPLC-grade or highest pure grade) were supplied by BDH Chemicals Company Limited, Poole, Dorset, UK. Polyvinylidene fluoride (PVDF) membrane filters (13 mm diameter with 0.45 µm pore size, 13 mm diameter with 0.22 µm pore size, and 33 mm diameter with 0.22 μm pore size) were purchased from Merck KGaA, Darmstadt, Germany.

### 4.2. POW9^TM^ Product

POW9^TM^ (Lot. Number G&B/L3/0004) is a proprietary botanical formulation developed by G&B Enzymes Company Limited, Bang Bon, Bangkok, Thailand. The product (600 mg per capsule) consisted of extracts derived from nine medicinal plants traditionally associated with antioxidant and health-promoting activities, including turmeric *Curcuma longa* L. rhizome powder, cinnamon (*Cinnamomum verum*) bark, emblica (*Phyllanthus emblica* L.) fruit, ginkgo (*Ginkgo biloba* L.) leaves, gotu kola (*Centella asiatica*) leaves, black pepper (*Piper nigrum* L.) fruit, tea (*Camellia sinensis*) leaves, mangosteen (*Garcinia mangostana* L.) aril and roselle (*Hibiscus sabdariffa* L.) flowers. POW9^TM^ is commercially available in Thailand as a nutraceutical dietary supplement.

### 4.3. Preparation of POW9^TM^ Solution

POW9^TM^ was prepared as a homogeneous suspension rather than a fully dissolved solution, reflecting the complex botanical nature of the formulation. The powder was dissolved in a culture medium containing 0.01% (*v*/*v*) Tween 20, then vortexed thoroughly, and sonicated for 20 min to ensure uniform dispersion. The POW9^TM^ stock solution, with a final concentration of 100 mg/mL, was filtered through the syringe PVDF membrane. Sterilization was performed after complete dispersion, and no visible residue remained on the membrane filter, indicating effective passage of suspended material under the applied conditions. The POW9^TM^ working solution was freshly prepared immediately before each experiment by diluting the stock solution with 0.1% Tween 20 solution and was not stored for reuse. Although formal chemical stability studies were not conducted, the formulation was handled consistently across experiments, and treatment media were used within the same experimental timeframe as the in vitro assays.

### 4.4. UHPLC-ESI-QTOF-MS Profiling of Phytocompounds

Chemical constituents in POW9^TM^ were analyzed using a powerful UHPLC-ESI-QTOF-MS technique as previously described by Hodgson and colleagues [22] with slight modifications [23]. The instrument system was composed of an HPLC machine (Agilent 1260 Infinity II LC, Agilent Technologies, Inc., Santa Clara, CA, USA) equipped with an electrospray ionization (ESI)-QTOF-MS. In the MS system, nitrogen gas nebulization was set at 45 pounds per square inch (psi) with a flow rate of 5.0 L/min at 300 °C. The sheath gas was set at 11.0 L/min at 250 °C, and the capillary and nozzle voltage values were set at 3.5 kV and 500 V, respectively. A complete mass scan was conducted with mass-to-charge ratio (*m*/*z*) values ranging from 200 to 3200. All operations, acquisitions, and analyses of the data were monitored using Agilent UHPLC-QTOF-MS MassHunter Acquisition Software Version B.04.00, with the “Find by Be” algorithm, to generate a list of precise mass match compounds. Peak identification was performed in positive modes using the library database, and the identification scores were further selected for characterization and *m*/*z* verification.

In analysis, 500 mg of the POW9^TM^ sample was reconstituted in 1% (*v*/*v*) DMSO (1.0 mL), ultrasonicated on an ice bath for 10 min, and then centrifuged at 17,000× *g* at room temperature for 10 min. Afterward, the supernatant was passed through a syringe filter (cellulose ester type, 13 mm diameter, 0.45 µm pore size, Merck Amicon filter, Sigma-Aldrich Chemical Company, Limited, Saint Louis, MO, USA). In the analysis, the filtrate (5 μL) was injected into the HPLC system using an autosampler and fractionated on a column (InfinityLab Poroshell 120 EC octadecyl silane type, 2.1 mm × 100 mm, 2.7 µm particle size, Agilent Technologies Company, Santa Clara, CA, USA) that had been thermally regulated at 40 °C and eluted in a linear gradient mode using mobile phase A (0.1% formic acid in DI) and mobile phase B (0.1% formic acid in acetonitrile) at a flow rate of 0.35 mL/min for 60 min. The timing program employed for gradient elution was as follows: 0 ⟶ 15 min; %A/B (100/0 ⟶ 90/10), 15 ⟶ 30 min; %A/B (90/10 ⟶ 40/60); 30 ⟶ 45 min: A (40/60 ⟶ 10/90); and 45 ⟶ 60 min; A (10/90 ⟶ 0/100). Peak identification was performed in positive mode using the library database, and the identification scores were sorted for characterization and m/z verification purposes.

Mass error in parts per million (ppm) was calculated using the Formula (1):

(1) $$\mathrm{Mass}\;\mathrm{error}\;(\mathrm{ppm})\;=\;(\mathrm{Observed}\;\mathrm{mass}\;-\;\mathrm{Calculated}\;\mathrm{mass})/\mathrm{Calculated}\;\mathrm{mass}\;\times \;10^{6}$$

Compound annotation was performed based on accurate mass matching with a tolerance threshold of ±10 ppm, in combination with isotopic pattern consistency and database/library comparisons.

### 4.5. Estimation of TPC and TFC

TPC was determined using a colorimetric method [30]. POW9^TM^ solution (100 µL) was mixed with 10% (*v*/*v*) Folin–Ciocalteu reagent (200 µL) and 10% (*w*/*v*) sodium carbonate (800 µL). The mixture was incubated at 25 °C for 30 min, and the absorbance (A) value was measured at a wavelength of 700 nm against a reagent blank using a double-beam ultraviolet–visible (UV-Vis) spectrophotometer (Model UV-1800, Shimadzu Corporation, Kyoto, Japan). TPC was determined from a standard curve of GA and reported in terms of mg GA/g.

TFC was determined using the colorimetric method based on the formation of a stable complex between aluminum ions and flavonoids [37]. The POW9^TM^ solution (250 µL) was mixed with a chromogenic reagent containing 10% (*w*/*v*) aluminum chloride (50 µL), 1 M potassium acetate (50 µL), and DI (2.15 mL). The mixture was then incubated in the dark at 25 °C for 30 min, and the A value was measured at a wavelength of 415 nm against a reagent blank using a UV-Vis spectrophotometer [38]. TFC was determined from a standard curve of Q and reported as mg QE/g.

### 4.6. DPPH Radical Scavenging Activity Assay

The free radical scavenging activity of POW9^TM^ was determined based on a DPPH colorimetric method [31]. In the assay, 40 μL of POW9^TM^ solution (5, 10, and 20 mg/mL) was mixed with 160 μL of freshly prepared 200 μM DPPH solution and incubated at 37 °C for 30 min. Following incubation, the A value was measured using a microplate reader (BioTek Lonza ELx808LBS, BioTek Instruments, Saint Winooski, VT, USA) at 517 nm. Antioxidant activity was calculated using the Formula (2) [32] as follows:

(2) $$\mathrm{Inhibition}\;\mathrm{of}\;\mathrm{DPPH}∙\;\mathrm{generation}\;=\;(A_{c}\;-\;A_{t})/A_{c}\;\times \;100$$

where A_c_ = absorbance of controls and A_t_ = absorbance of treatments. Accordingly, IC_50_ values for POW9^TM^ were calculated using GraphPad Prism software version 7.0 (Informer Technologies, Inc., Los Angeles, CA, USA).

### 4.7. Assessment for Effect of POW9^TM^ on Vera and Breast Cancer Cells

#### 4.7.1. Cell Culture

Vero cell line (ATCC-CCL-81™) obtained from the American Type Culture Collection (Manassas, VA, USA) were cultured in DMEM (high glucose and L-glutamine.) supplemented with 10% (*v*/*v*) heat-inactivated FBS, 1% penicillin–streptomycin (100 U/mL penicillin and 100 µg/mL streptomycin) at 37 °C in a humidified atmosphere containing 5% CO_2_. When reaching 80–90% cell confluency, the medium was discarded, the cell monolayer was gently washed once with sterile PBS without calcium and magnesium and detached from the plate by incubating with 0.25% (*w*/*v*) T-EDTA briefly until cell detachment was observed. The cell suspension was neutralized with complete growth medium and collected by gentle pipetting. Afterward, the cells were sedimented by centrifugation at 200× *g* for 5 min, resuspended in fresh complete medium and seeded into new culture vessels at the desired density. Medium was replaced every 2–3 days, and cell morphology and confluency were monitored regularly using phase-contrast microscopy. All cell culture procedures were performed under aseptic conditions in a Class II biosafety cabinet.

MCF-7 (ATCC HB-22™) and MDA-MB-231 (HTB-26™), obtained from the ATCC, were cultured according to the method established by Gest and coworkers [33]. RPMI 1640 medium was supplemented with 10% (*v*/*v*) FBS, 2 mM glutamine, 100 IU/mL penicillin and 100 μg/mL streptomycin while DMEM supplemented with 10% (*v*/*v*) FBS, 4 mM glutamine, 100 U/mL penicillin and 100 μg/mL streptomycin, and the media were sterilized by ultra-filtration through the membrane (33 mm diameter, 0.22 μm pore size). Briefly, MDA-MB-231 cells (3 × 10^4^/well) were grown in the RPMI 1640 medium and MCF-7 cells (3 × 10^4^/well) were grown in the DMEM at 37 °C in a humidified 5% CO_2_ atmosphere till achieved 80% cell confluence was achieved.

#### 4.7.2. Investigation of Antiproliferative Activity of POW9™

In the study, MCF-7 and MDA-MB-231 cell cultures were incubated with POW9^TM^ solution (1.56–100 mg/mL) at 37 °C for 72 and 96 h. After incubation, the cells were washed two times with PBS and determined cell viability using the MTT method, as described below. The 0-concentration group represents the vehicle (untreated) control and consisted of complete culture medium containing the same final concentration of Tween 20 as used in POW9™-treated samples, but without POW9™. The concentration of Tween 20 used was low and did not induce detectable cytotoxic effects under the experimental conditions. All viability measurements were normalized to this vehicle control to exclude potential effects of the solvent. All cell-based experiments were performed using three independent biological replicates, defined as separate experiments conducted on different days with independently cultured cells and freshly prepared POW9™ solutions. Within each biological replicate, MTT measurements were performed in technical triplicate.

#### 4.7.3. Assay of Cell Viability

Colorimetric MTT assay was performed to evaluate the cell toxicity of POW9^TM^, following the method described by Nguyen and coworkers [35]. The treated cells were incubated with 1 mg/mL MTT solution (20 μL) at 37 °C for 4 h. Following incubation, the produced blue-colored formazan crystal was solubilized with 1% DMSO solution (200 μL) with shaking for 1 h at room temperature [36]. The A was recorded at 570 nm using a BioTek 96-well microplate reader. The percentage of cell viability was calculated using the Formula (3) as follows:

(3) $$\mathrm{Percentage}\;\mathrm{of}\;\mathrm{cell}\;\mathrm{viability}\;=\;(A_{c}\;-\;A_{t})/A_{c}\;\times \;100$$

where A_c_ = absorbance of untreated controls, A_t_ = absorbance of treatments. Accordingly, IC_50_ values for each extract were calculated using the GraphPad Prism software version 7.0 (GraphPad Software Inc., San Diego, CA, USA).

### 4.8. Statistical Analysis

The in vitro cell culture experiments were conducted in triplicate. The biochemical assays were also performed in triplicate. The data sets were statistically analyzed using the Statistical Package for the Social Sciences (SPSS) Statistics for Windows version 22 Program (IBM Corporation, Armonk, NY, USA) and expressed as values of mean ± standard deviation (SD). The statistical differences were tested using the One-Way Analysis of Variance (ANOVA), followed by the post hoc analysis via Tukey–Kramer test, which *p* value < 0.05 was considered statistically significant. Prior to performing the ANOVA test, data were examined for approximate normality and homogeneity of variance to verify that ANOVA assumptions were met. Dose–response curves were fitted using nonlinear regression analysis based on a four-parameter logistic model, and IC_50_ (or LC_50_, where applicable) values were calculated using SigmaPlot version 15.0 Program (SYSTAT Software Inc., Grafiti, St. Palo Alto, CA, USA). These IC_50_ values served as the primary quantitative endpoint for cytotoxicity assessment.”

## 5. Conclusions

This study provides an untargeted UHPLC-ESI-QTOF-MS chemical profile of POW9™ and a comparative in vitro assessment of its effects on cell viability in Vero, MCF-7, and MDA-MB-231 cells. POW9™ showed concentration- and time-dependent antiproliferative effects in breast cancer cell lines under the tested conditions, while exhibiting lower cytotoxic effects in Vero cells. The antioxidant assays (TPC, TFC, TEAC) are presented as formulation-level compositional characterization and do not establish an anticancer mechanism. Overall, the findings should be interpreted as exploratory and support further work, including mechanistic cellular assays, benchmarking against reference compounds, and in vivo evaluation to determine translational relevance.

## Figures and Tables

**Table 1:** Polar phenolic compounds detected in POW9^TM^ product by UHPLC-ESI-QTOF-MS.

(**A**) Negative mode [M-H]^−^							
**T_R_** (**min**)	**Mass** (**g/mol**)		**Error** (**ppm**)	**Calculated (ppm)**	**Mass Difference (g/mol)**	**Molecular Formula**	**Possible Compounds**
	**Reference**	**Observed**					
41.699	216.277	216.136	0.34	−651.9	0.141	C_11_H_20_O_4_	Butyl butyryl lactate
41.906	294.386	294.183	−0.30	−689.6	0.203	C_17_H_26_O_4_	Myrsinone
42.198	372.458	372.214	0.90	−655.1	0.244	C_19_H_32_O_7_	Blumenol C glucoside
42.417	197.320	197.178	−0.36	−719.6	0.142	C_12_H_23_NO	N-Methylundec-10-enamide
42.936	432.50	432.236	−0.21	−610.4	0.264	C_21_H_36_O_9_	Glucosyl dihydroxyfarnesadienoate
43.254	198.161	198.162	0.26	5.0	0.001	C_12_H_22_O_2_	Citronellol acetate
43.554	326.495	326.191	0.62	−931.1	0.304	C_18_H_30_O_3_S	2-Dodecylbenzenesulfonic acid
44.359	244.311	244.167	0.48	−589.4	0.144	C_13_H_24_O_4_	1,4-Nonanediol diacetate
44.888	256.381	256.204	0.74	−690.4	0.177	C_15_H_28_O_3_	Ipomea tetrahydrofuran
45.047	226.34	226.103	−1.66	−1047.1	9.237	C_12_H_18_O_2_S	Methyl-3-furanyl thiol-4-heptanone
45.220	258.36	258.183	−0.38	−685.2	0.177	C_14_H_26_O_4_	Diethyl decanedioate
45.541	270.413	270.219	0.12	−717.2	0.194	C_16_H_30_O_3_	16-Hydroxy-9E-hexadecenoic acid
45.913	272.38	272.198	1.14	−668.2	0.182	C_15_H_28_O_4_	7-Bisabolene-2,3,10,11-tetrol
45.981	288.42	288.230	−0.5	−658.8	0.190	C_16_H_32_O_4_	9,10-Dihydroxy-hexadecanoic acid
46.195	358.47	358.272	0.63	−552.4	0.198	C_20_H_38_O_5_	Dimethoxy-13-hydroxy-10-octadecenoic acid
46.276	278.34	278.152	0.62	−675.4	0.188	C_16_H_22_O_4_	Diisobutyl phthalate
46.355	316.167	316.167	0.72	0.0	0.000	C_19_H_24_O_4_	Isopulegone caffeate
46.516	356.5	356.292	2.08	−583.4	0.208	C_21_H_40_O_4_	Glyceryl monoleate
46.538	251.32	251.152	0.88	−668.7	0.168	C_14_H_21_NO_3_	Furmecyclox
46.547	298.5	298.251	0.06	−834.2	0.249	C_18_H_34_O_3_	Ricinoleic acid
46.632	272.424	272.235	0.60	−693.8	0.198	C_16_H_32_O_3_	2-Hydroxy palmitic acid
46.897	328.49	328.261	1.25	−697.1	0.229	C_19_H_36_O_4_	Avocadene 4-acetate
47.051	312.47	312.176	0.32	−940.9	0.294	C_17_H_28_O_3_S	N-Undecylbenzenesulfonic acid
47.215	342.50	342.277	−0.02	−651.2	0.223	C_20_H_38_O_4_	Eicosanedioic acid
47.720	334.40	334.143	−0.98	−768.5	0.257	C_11_H_22_N_6_O_4_S	Arg-Gly-Cys
47.889	284.48	284.272	−0.36	−731.1	0.208	C_18_H_36_O_2_	Lambda Isostearic acid
47.935	296.47	296.181	0.99	−974.8	0.289	C_17_H_28_O_2_S	S-Farnesyl thioacetic acid
48.520	412.655	412.355	1.33	−727.9	0.300	C_25_H_48_O_4_	2,3-Dihydroxypropyl docosenoate
48.789	406.56	406.272	0.75	−708.3	0.288	C_24_H_38_O_5_	7-Ketodeoxycholic acid
48.853	280.452	280.240	0.50	−756.1	0.212	C_18_H_32_O_2_	Octadecadienoic acid
(**B**) Positive mode [M-H]^+^							
**T_R_** (**min**)	**Mass** (**g/mol**)		**Error** (**ppm**)	**Calculated (ppm)**	**Mass Difference (ppm)**	**Molecular Formula**	**Possible Compounds**
	**Reference**	**Observed**					
30.614	440.530	440.218	−1.05	−708.3	0.312	C_22_H_28_N_6_O_4_	Trp-His-Val
37.256	508.770	508.414	−1.91	−699.8	0.356	C_31_H_56_O_5_	1,3-Ditetradecenoin
42.966	278.260	278.101	−2.17	−571.4	0.159	C_11_H_18_O_8_	6-O-(4-Hydroxy-2-methylene-butanoyl)-β-D-glucopyranose
42.984	165.15	165.043	−1.68	−648.0	0.107	C_8_H_7_NO_3_	6-Methoxy-2-benzoxazolone
43.104	414.502	414.216	−2.10	−690.1	0.286	C_23_H_30_N_2_O_5_	Desacetylvindoline
44.026	300.475	300.259	1.45	−718.8	0.216	C_18_H_32_O_3_	9(S)-Hydroxyoctadecenoic acid
44.318	437.550	437.291	−1.46	−592.	0.259	C_21_H_44_NO_6_P	1-Palmitoyl-sn-glycero-3-phosphatidal ethanolamine
45.023	220.22	220.106	1.33	−517.6	0.114	C_8_H_16_N_2_O_5_	Thr-Thr
45.371	326.250	326.064	−0.43	−570.3	0.186	C_14_H_14_O_9_	Fertaric acid
46.223	98.10	98.037	−1.17	−642.2	0.063	C_5_H_6_O_2_	β-Vinyl acrylic acid
46.275	371.480	371.188	1.84	−785.8	0.292	C_25_H_25_NO_2_	6-Methoxyindole analog
46.302	320.410	320.152	−0.32	−804.9	0.258	C_12_H_24_N_4_O_4_S	Ala-Lys-Cys
47.152	177.160	177.064	−1.77	−542.0	0.096	C_6_H_11_NO_5_	3-Keto-scyllo-inosamine
48.115	678.426	678.426	0.64	0.0	0.000	C_38_H_63_O_8_P	[3-Phosphonooxy-2-tridecanoyloxypropyl]-docosahexaenoate
48.182	381.49	381.265	−2.17	−589.9	0.225	C_18_H_40_NO_5_P	Sphinganine phosphate
49.431	272.38	272.199	0.40	−664.5	0.181	C_15_H_28_O_4_	7(14)-Bisabolene-2,3,10,11-tetrol
50.549	126.111	126.031	3.53	−634.5	0.080	C_6_H_6_O_3_	Tamarindienal
50.555	294.11	294.110	0.36	0.0	0.000	C_15_H_18_O_6_	Cyclocalopin F
50.559	452.531	452.237	0.75	−649.7	0.294	C_25_H_34_F_2_O_5_	Tafluprost

**Table 2:** TPC, TFC and antioxidant activity of POW9^TM^ product. Data are presented as mean ± standard deviation (SD) of three independent analyses.

Compound	TPC	TFC	TEAC
POW9	190.3 ± 3.5 mg GAE/g	115.2 ± 1.5 mg QE/g	33.73 mg of Trolox/g

Abbreviation: TEAC = Trolox equivalent antioxidant capacity, TFC = total flavonoid content, TPC = total phenolic product.

## Data Availability

The original contributions presented in this study are included in the article and Supplementary Materials. Further inquiries can be directed to the corresponding authors.

## Supplementary Materials

The following supporting information can be downloaded at: https://www.mdpi.com/article/10.3390/ijms27031246/s1.

## Abbreviations

The following abbreviations/symbols are used in this manuscript:

A	Absorbance
ABTS	2,2′-Azino-bis(3-ethylbenzothiazoline-6-sulfonic acid)
ANOVA	Analysis of Variance
DMEM	Dulbecco’s modified Eagle medium
DMSO	Dimethyl sulfoxide
DPPH	2,2-Diphenyl-1-picrylhydrazyl
ER	Estrogen receptor
ESI	Electrospray ionization
FBS	Fetal bovine serum
GA	Gallic acid
GAE	Gallic acid equivalent
HER2	Human epidermal growth factor receptor 2
IC_50_	Half-maximal inhibitory concentration
MTT	3-(4,5-Dimethylthiazol-2-yl)-2,5-diphenyltetrazolium bromide
*m*/*z*	Mass-to-charge ratio
PBS	Phosphate-buffered saline
psi	pounds per square inch
PVDF	Polyvinylidene fluoride
Q	Quercetin
QE	Quercetin equivalent
TEAC	Trolox equivalent antioxidant capacity
T-EDTA	Trypsin-ethylene diaminetetraacetic acid
TFC	Total flavonoid content
TNBC	Triple-negative breast cancer
TPC	Total phenolic content
Trolox	6-Hydroxy-2,5,7,8-tetramethylchroman-2-carboxylic acid
UHPLC-ESI-QTOF-MS	Ultrahigh performance liquid chromatography-electrospray ionization-quadrupole time-of-flight mass spectrometer
*v*/*v*	Volume by volume
[M+H]^+^	Protonated molecule
[M-H]^−^	Deprotonated molecule
