# Supplementary material for: UHPLC-QTOF-MS Profiling of Chemical Constituents in POW9TM Cocktail with Antioxidant and Anti-Proliferative Potentials Against Vero, MCF-7 and MDA-MB-231 Cells
Source: Int J Mol Sci. 2026 Jan 27;27(3):1246. doi: 10.3390/ijms27031246 (PMC12897745; doi:10.3390/ijms27031246)
Supplement: Supplementary file 1 [file ijms-27-01246-s001.zip › ijms-4083430-supplementary.pdf]

International Journal of
*Molecular Sciences*

MDPI

# Supplementary Figures

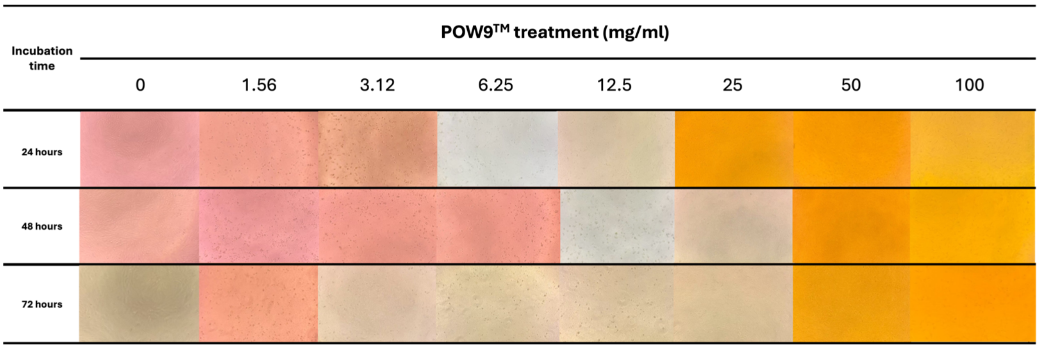


**Figure S1.** Microscopic illustration for viability of Vera cells treated with POW9™ (0-100 mg/mL) for 24, 48 and 72 hours.

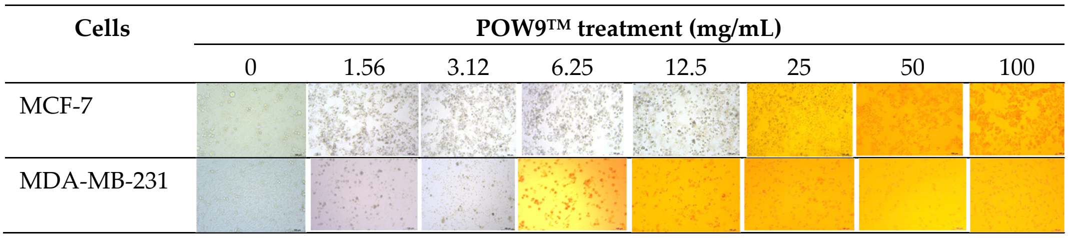


**Figure S2.** Microscopic illustration for viability of MCF-7 and MDA-MB-231 cells treated with POW9™ (0-100 mg/mL) for 72 hours.

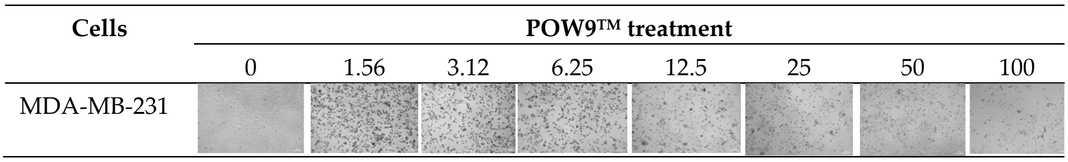


**Figure S3.** Microscopic illustration for viability of MDA-MB-231 cells treated with POW9™ (0-100 mg/mL) for 96 hours.
